# Supplementary figures and images for: Centriolar satellites assemble centrosomal microcephaly proteins to recruit CDK2 and promote centriole duplication
Source: eLife. 2015 Aug 22;4:e07519. doi: 10.7554/eLife.07519 (PMC4574112; doi:10.7554/eLife.07519)

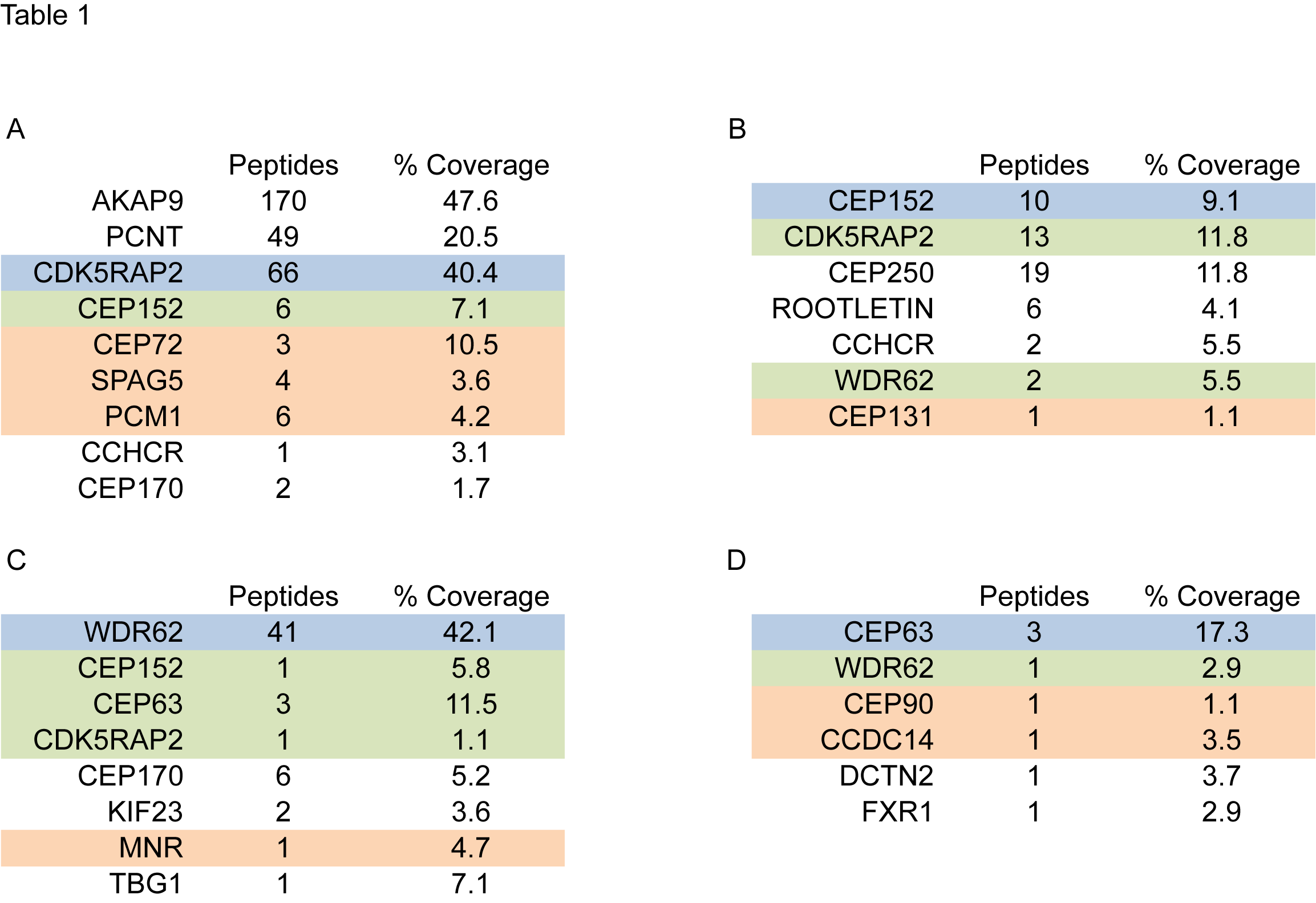

Supplement: Supplementary file 1. — CDK5RAP2, CEP152, WDR62 and CEP63 mass spectrometry analysis. (A–D) Selected list of CDK5RAP2, CEP152, WDR62, and CEP63 interacting centrosomal proteins, including peptide counts and percent coverage. Coprecipitating proteins were counterscreened against c-Myc and FLAG interacting proteins, which served as negative controls. We used the proteomic analysis by Andersen et al. (2003) and Jakobsen et al. (2011) to suggest the identity of centrosomal proteins for additional analysis. Precipitated proteins are highlighted in blue, MCPH-associated proteins are in green, and centriolar satellite proteins are in orange. DOI: http://dx.doi.org/10.7554/eLife.07519.035 [file elife07519s001.tiff]
