# Supplementary material for: Centriolar satellites assemble centrosomal microcephaly proteins to recruit CDK2 and promote centriole duplication
Source: eLife. 2015 Aug 22;4:e07519. doi: 10.7554/eLife.07519 (PMC4574112; doi:10.7554/eLife.07519)
Supplement: Supplementary file 2. — siRNA sequences. Human Stealth siRNAs to CDK5RAP2, WDR62, CEP63, SPAG5, CEP72, CEP131, MNR, CEP90 and CCDC14 were obtained from Life Technologies. SC, CEP152, CEP63 NB and CEP215 siRNAs were synthesized by Life Technologies. DOI: http://dx.doi.org/10.7554/eLife.07519.036 [file elife07519s002.pdf]

Table 2:

Gene	Target sequences of siRNA
CDK5RAP2 #1	GGTTGGGAAATGGTCTGCTCCCAAA
CDK5RAP2 #2	GAGCAAGCCCAAGAATGGTTCTGAT
CEP215 282	UUCAUUUCUAAGAUCACGGAU (Graser et al., 2007)
CEP215 283	UUUAGUUAGGAGAUCUUCCAC (Graser et al., 2007)
CEP152 #1	CAGCTCTTTGAGGCTTATGAG (Cizmecioglu et al., 2010)
CEP152 #2	GCGGATCCAACTGGAAATCTA (Cizmecioglu et al., 2010)
WDR62 #1	CCAACTGCATGAAGCAGCACTTGCT
WDR62 #2	AGCAAGTGCTGCTTCATGCAGTTGG
CEP63 #1	CCATGGAATATAAGCAGGAGTTGAA
CEP63 #2	CAGGGAAGATCGGTCTGAAATTGAG
CEP63 NB	AGUCCAGCGAUUUCUGACG (Brown et al., 2013)
CEP72 #1	CCGGAAAGTGCAGGAAGCGAAGAAT
CEP72 #2	GCTCTGGAGAGTAAGTCCCTGCAAA
SPAG5	GCAGCAACAAGACATGATA (Liu et al., 2009)
CEP131 #1	CCAGGGAGACCAGAGTGCCAGGAAT
CEP131 #2	GCCACCCACTCAGCCCGGAACAATA
CCDC14 #1	CCACAACAAGGAATTCATAAGGAAA
CCDC14 #2	TGGCAACAGTCAGTTTGCATCACAA
MNR #1	GCAGACAACAGTTTCATCCAGATTA
MNR #2	CCAGGAGTCTGTTCGTCAAAGATAT
CEP90 #1	TCTGCAAGAGGAAACAGCAAGAAAT
CEP90 #2	AGGAAATCTGTGAACTACAAGTGAA
Scrambled control	AAACTAAACTGAGGCAATGCC

References:

Cizmecioglu, O., Arnold, M., Bahtz, R., Settele, F., Ehret, L., Haselmann-Weiss, U., Antony, C., and Hoffmann, I. (2010). Cep152 acts as a scaffold for recruitment of Plk4 and CPAP to the centrosome. J Cell Biol *191*, 731-739.

Liu, L., Akhter, S., Bae, J.B., Mukhopadhyay, S.S., Richie, C.T., Liu, X., and Legerski, R. (2009). SNM1B/Apollo interacts with astrin and is required for the prophase cell cycle checkpoint. Cell Cycle *8*, 628-638.

Graser, S., Stierhof, Y.D., and Nigg, E.A. (2007). Cep68 and Cep215 (Cdk5rap2) are required for centrosome cohesion. J Cell Sci *120*, 4321-4331.

Brown, N.J., Marjanovic, M., Luders, J., Stracker, T.H., and Costanzo, V. (2013). Cep63 and cep152 cooperate to ensure centriole duplication. PLoS One *8*, e69986.
